# Can body appreciation protect against media-induced body dissatisfaction?

**DOI:** 10.1186/2050-2974-3-S1-O19

**Published:** 2015-11-23

**Authors:** Rachel Andrew, Marika Tiggemann, Levina Clark

**Affiliations:** 1 School of Psychology, Flinders University, Australia

## 

The study examined the protective role of body appreciation against body dissatisfaction (a known risk factor for disordered eating), resulting from thin-ideal exposure. We also tested potential mediating mechanisms, specifically, self-objectification and appearance comparison, and explored strategies that women may use to protect their body image.

University women (N = 68) first completed measures of body appreciation and media protective strategies. During a subsequent session, participants viewed 11 thin-ideal advertisements. Pre and post advertisement exposure body dissatisfaction was measured, followed by state assessments of self-objectification, appearance comparison and media protective strategies.

Results showed that body appreciation predicted change in body dissatisfaction. Specifically, participants with low body appreciation reported increased body dissatisfaction after exposure, whereas participants with high body appreciation did not experience an increase. Neither state self-objectification nor state appearance comparison accounted for body appreciation's protective effect against thin-ideal induced body dissatisfaction. Trait and state media protective strategies were also found to be positively related with body appreciation.

The results indicate that body appreciation is able to some extent shield young women against body dissatisfaction resulting from acute exposure to thin-ideal media. The findings have practical implications for interventions aimed at body dissatisfaction and disordered eating.

